# A Cross‐Sectional Survey of Orthodontic Retention Practices and Opinions Among General Dental Practitioners in Australia

**DOI:** 10.1002/cre2.70317

**Published:** 2026-02-23

**Authors:** Maurice J. Meade, Craig Dreyer

**Affiliations:** ^1^ PR Begg Chair in Orthodontics, Orthodontic Unit, School of Dentistry Adelaide University Adelaide Australia; ^2^ Orthodontic Unit, School of Dentistry Adelaide University Adelaide Australia

## Abstract

**Objective:**

To investigate orthodontic retention opinions and practices of general dental practitioners (GDPs) in Australia.

**Materials and Methods:**

A piloted electronic survey was distributed to dentists via Australian Dental Association communication channels. Survey questions related to respondent demographics, opinions regarding preferred retainer choice and protocols, factors concerning retainer choice and the management of retainers.

**Results:**

Data from 683 GDP respondents were received and analyzed. Most (*n* = 351; 51.4%) indicated that they offered no orthodontic treatment services whereas 332 (48.6%) offered limited or comprehensive orthodontics. The clear plastic retainer and bonded retainer (BR) combined was most prescribed by GDPs in the maxilla (40.0%–45.5%) and mandible (40.6%–52.4%). GDPs who offered orthodontic services were more likely (*p* < 0.01) to always or mostly replace or repair (*p* < 0.01) BRs than those who did not. Between 57.8% and 61.2% of respondents were very or somewhat concerned that BRs may be associated with periodontal problems over the long term. Most (*n* = 445; 83%) indicated that they would like the treating orthodontist to inform them that treatment had been completed. Between 68.1% and 72.2% would like to be informed by the orthodontist about patients' retention protocols. Respondents who offered orthodontic services were more likely to recommend patients bring their removable retainers to review or treatment appointments (*p* < 0.01).

**Conclusion:**

Differences in opinion regarding the management of the retention phase between GDPs in Australia who offered orthodontic services and those who did not were observed. It is essential that the orthodontic profession develop guidance and education regarding the management of the retention phase.

## 1 Introduction

Following the completion of active orthodontic therapy, patients are routinely prescribed retainers (Al‐Moghrabi, Littlewood et al. 2021). These aim to minimize treatment relapse (Martin et al. 2023) which is the return to the traits of the pretreatment malocclusion following orthodontic correction (Meade and Millett 2020). Some malocclusion characteristics (such as midline diastema and severely rotated teeth) may be comparatively more likely to relapse. In addition, relapse has also come to include the normal intra‐ and inter‐arch occlusal changes that occur with continued facial growth (Millett 2021).

Retainers can either be removable or fixed with many orthodontists prescribing a combination of both (Martin et al. 2023; Meade and Millett 2020; Padmos et al. 2019). Each retainer type has its proposed advantages and disadvantages (Meade and Millett 2020, 2015; Alkadhimi and Sharif 2019). Potential problems associated with removable retainers (RRs) include loss and the reliance on patient adherence with wear protocols (Meade and Millett 2015; Al‐Moghrabi et al. 2023). Disadvantages associated with bonded retainers (BRs) include bond failure and “Wire Syndrome,” a phenomenon in which unwanted tooth movement can occur even though the retainer wire remains attached or bonded to the tooth (Padmos et al. 2019; Meade and Millett 2020; Abu Arqub et al. 2023; Kučera et al. 2022; Katsaros et al. 2007; Charavet et al. 2022). The findings from several national investigations suggest that orthodontists routinely recommend the lifelong wear of retainers to maintain orthodontic treatment outcomes (Meade and Millett 2013; Carneiro et al. 2022; Meade and Dreyer 2019; Padmos et al. 2018). Previous surveys have also indicated that most orthodontists carry out retention or retainer checks for 1–2 years before discharging patients from their care (Meade and Millett 2013; Carneiro et al. 2022; Meade and Dreyer 2019; Saw et al. 2025). Consequently, general dental practitioners (GDPs) may be required to oversee the post‐orthodontic treatment retention phase or manage associated problems such as retainer loss or breakage or retainer related harm including inadvertent tooth movement (Abu Arqub et al. 2023; Schreuder et al. 2025; Molyneaux et al. 2021; Rafflenbeul et al. 2021; Habegger et al. 2017).

Therefore, effective communication between the orthodontist and the GDP, in addition to adequate relevant knowledge among GDPs, is essential to ensure optimal care for patients during the retention phase. Surveys of GDPs regarding retention have been undertaken in several countries (Schreuder et al. 2025; Molyneaux et al. 2021; Rafflenbeul et al. 2021; Habegger et al. 2017; Kotecha et al. 2015; Arnold et al. 2014) and appear to have focused on GDP opinions and protocols regarding BRs (Schreuder et al. 2025; Rafflenbeul et al. 2021; Habegger et al. 2017) or aimed to determine GDP opinions about retention (Molyneaux et al. 2021; Kotecha et al. 2015). No study, however, has investigated GDP opinions on retention in Australia. Furthermore, data concerning the opinions of GDPs based on whether they practice orthodontic treatment and the retention practices of GDPs who carry out orthodontic treatment are limited (Schreuder et al. 2025; Meade and Weir 2024). The aim of the present study was to determine retention practices of GDPs and to compare opinions regarding orthodontic retention by GDPs who carry out orthodontic treatment with those who do not.

## 2 Methods

### 2.1 Ethical Approval

Ethical approval was granted by the University of Adelaide Human Research Ethics Committee [H‐2023‐294], following the granting of permission by the Australian Dental Association (ADA) to carry out the survey of their members.

### 2.2 Questionnaire Development

An anonymous cross‐sectional electronic questionnaire (e‐questionnaire) was created on the Qualtrics (Provo, Utah, USA) software platform. The development, oversight and reporting of the survey aimed to correspond with relevant published guidelines (Burns et al. 2008; Eysenbach 2004).

### 2.3 Pilot Questionnaire

A pilot e‐questionnaire was developed from previous surveys (Carneiro et al. 2022; Meade and Dreyer 2019) conducted of specialist orthodontists and included pre‐testing of GDP colleagues working in different practice environments.

### 2.4 Validity Testing

The validity of the questionnaire was established by distributing the pilot questionnaire and study information sheet to five GDPs working in two states in Australia. The GDPs were colleagues of the authors and had between 4 and 27 years in general dental practice experience. Two had never previously provided orthodontic treatment. The GDPs reported that the time to complete the questionnaire was less than 15 minutes. Also provided by the GDPs were comments concerning the clarity and pertinence of the survey questions. Test–retest reliability was determined using Cohen's kappa analysis of three GDPs repeating the survey after a 3‐week interval.

### 2.5 Readability Evaluation

The readability of the questions was assessed by the use of the Flesch–Kincaid Grade Level tool (Kincaid et al. 1975). A score of 6.5 suggested a basic to average reading level.

### 2.6 Content of the Finalized Questionnaire

The 25‐question survey comprised the following: Section Objective, 1 pertained to respondent demographic details including the extent to which respondents carried out orthodontic treatment. Section Materials and Methods, 2 contained questions concerning GDP opinions about the effectiveness of retainers at maintaining treatment results and how long retainers should be worn following orthodontic treatment. Section Results, 3 invited respondents to answer questions about the importance of a range of factors in the choice of a specific type of retainer. Section Conclusion, 4 related to questions about GDP comfort in the management of lost and broken retainers and communication protocols with specialist orthodontists.

### 2.7 Population and Sampling

The ADA is the primary representative organization for dentists in Australia. The requirements for potential inclusion in the survey were dentists who were registered with the Australian Health Practitioner Regulation Agency (AHPRA), and whose main workplace was in Australia. There were 24,722 individuals with general dental registration with AHPRA and approximately 13,100 non‐student members of the ADA at the time of the initial dissemination of the survey (Dental Board of Australia 2025; Australian Competition & Consumer Commission 2025). Dentists who were also registered as specialist dentists were excluded from survey analysis. Additionally, because of the nature of the dissemination of the questionnaire, non‐ADA members may have responded. If these respondents otherwise met selection criteria, their responses were included in the assessment.

### 2.8 Dissemination of the Survey

Information concerning the investigation and a link to the survey was distributed via state and federal ADA communication channels and through the eviDent Foundation Dental Practice‐Based Research Network (South Yarra, Victoria, Australia). Cookies on the web browsers of those who had responded were employed by the Qualtrics platform to reduce the risk of respondents responding to the survey more than once. The survey was open from September 1st to October 30th, 2024, with reminder correspondences being distributed on October 1st.

Responses from the survey were exported from the Qualtrics software platform to a Microsoft Excel (version 16.0; Microsoft, Redmond, WA, USA) spreadsheet for data cleaning and preliminary analysis. The responses from dentists who stated that they were also registered as specialist dentists were omitted from analysis.

### 2.9 Statistics

Descriptive statistics were computed by the GraphPad Prism (GraphPad Software Inc., La Jolla, CA, USA) statistical software package and provided in frequencies and percentages. Chi‐square tests ( *χ* ^2^) were carried out to determine whether there was a relationship in opinions between GDPs who offered orthodontic services, and those who did not. Statistically significant predictors of responses to selected questions following univariable/multivariable binary logistic regression were presented as odds ratios and their 95% confidence intervals.

A minimum sample size of 379 was required. This was based on the estimated 25,000 members of the ADA at the time of the investigation, a margin of error of 5% and a 95% confidence interval (Khanna et al. 2025).

## 3 Results

A total of 702 responses were recorded. This approximated to a response rate of 5.4% of the non‐student membership of the ADA. Following exclusion of the responses of 19 specialist dentists, data from 683 respondents were analyzed. Not all respondents answered all questions. Most (*n* = 355; 52.0%) respondents were male, followed by female (*n* = 319; 46.7%) and non‐binary/third gender/prefer not to say or did not answer (*n* = 9: 1.3%).

The number of years since graduation as a dentist for most respondents was either more than 30 (*n* = 199; 29.1%), 11–20 (*n* = 176; 25.8%) or 21–30 (*n* = 132; 19.3%). Ninety‐four (13.7%) respondents reported that it was between 6 and 10 years since dental graduation and 82 (12%) reported that it had been 0–5 years.

Most respondents indicated that they had obtained their dentistry degree or qualification from a university within Australia (*n* = 535; 80.6%). The United Kingdom (*n* = 29; 4.4%), New Zealand (*n* = 31; 4.7%) and “others” (*n* = 69; 10.4%) constituted the next most common places from which respondents reportedly obtained their dental qualification.

The majority (*n* = 568; 86%) of respondents worked only in private practice with 60 (9.1%) indicating that they worked only in public practice. Thirty‐two (4.8%) responded that they worked in a “public, private and/or university” clinical setting.

A total of 351 (51.4%) indicated that they offered no orthodontic treatment services whereas 194 (28.4%) offered limited orthodontic services and 138 (20.2%) offered comprehensive orthodontic services.

Table 1 shows that the clear plastic retainer (CPR) and the BR combined was the most prescribed retainer by GDPs following clear aligner therapy (CAT) and fixed appliance therapy (FAT). Of the 319 respondents who did not offer orthodontic services, 190 (59.6%) believed that the CPR was generally the most effective at maintaining end of orthodontic treatment results whereas 65 (20.3%) believed that the CPR and BR combined was generally most effective.

**Table 1:** Most common retainer prescribed following orthodontic treatment by GDPs who offered orthodontic services.

	Appliance							
	FAT				CAT			
	*N* = 185				*N* = 242			
Retainer	Max		Man		Max		Man	
	*N*	%	*N*	%	*N*	%	*N*	%
HR only	7	3.8	1	0.5	3	1.2	6	2.5
CPR only	70	37.8	66	35.7	102	42.1	90	37.2
BR only	18	9.7	39	21.1	19	7.9	13	5.4
BR and HR	2	1.1	1	0.5	1	0.4	2	0.8
CPR and BR	74	40.0	75	40.6	110	45.5	127	52.4
Other	14	7.6	3	1.6	7	2.9	4	1.7

*Note:* HRs and CPRs are removable retainers.

Abbreviations: %, percentage; BR, bonded retainer; CAT, clear aligner therapy; CPR, clear plastic retainer; FAT, fixed appliance therapy; GDP, general dental practitioner; HR, Hawley retainer; Man, mandible; Max, maxilla; N, number; N/A, not applicable.

**Figure 1:**
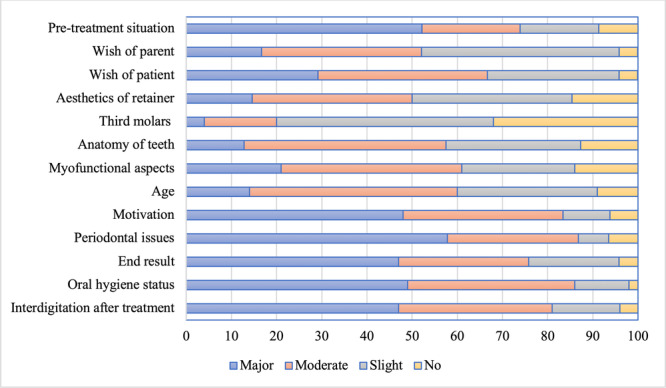
Importance of factors in the choice of specific retainers by percentage GDP (*n* = 644).

Figure 1 shows that 74.8% of respondents indicated that the pre‐treatment situation was a majorly or moderately important factor in the choice of retainer whereas just 20% (*n* = 136) stated that third permanent molars were a majorly or moderately important factor in the choice of a retainer.

Table 2 shows that the majority of respondents were happy to replace RRs in the event of loss or breakage whereas those who offered orthodontic services were significantly more likely to always or mostly repair RRs in the event of breakage (*χ* ^2^ = 39.7, *p* < 0.01). Table 3 shows that GDPs who offered orthodontic services, however, were significantly more likely to always or mostly replace (*p* < 0.01) or repair (*p* < 0.01) BRs than those who did not offer orthodontic services.

**Table 2:** Frequency of preparedness of GDPs to repair and replace RRs in the event of breakage or loss.

Replace/repair	GDP category	Frequency											
		Always		Mostly		Sometimes		Rarely		Never		Total	
		*N*	%	*N*	%	*N*	%	*N*	%	*N*	%	*N*	%
Replace	Ortho	125	42.8	62	21.2	91	31.2	9	3.1	5	1.7	292	100
	Non‐ortho	55	18.1	82	27.4	96	31.7	13	4.3	56	18.5	302	100
	Total	180	30.3	144	24.2	187	31.5	22	3.7	61	10.3	594	100
		*χ* ^2^ = 44.1, *p* < 0.01.^a^											
Repair	Ortho	93	32.1	64	22.1	84	28.9	30	10.3	19	6.6	290	100
	Non‐ortho	29	9.7	44	14.7	134	44.8	18	6.1	74	24.7	299	100
	Total	122	20.7	108	18.3	218	37.1	48	8.1	93	15.8	589	100
		*χ* ^2^ = 39.7, *p* < 0.01.^a^											

Abbreviations: *χ* ^2^, Chi‐square; GDP, general dental practitioner; N, number; Non‐ortho, GDPs who do not offer orthodontic services; Ortho, GDPs who offer orthodontic services; RR, removable retainer.

^a^ *χ* ^2^ test comparing ortho and non‐ortho according to almost/mostly versus rarely/never.

**Table 3:** Frequency of preparedness of GDPs to repair and replace BRs in the event of breakage or loss.

Replace/repair	GDP category	Frequency											
		Always		Mostly		Sometimes		Rarely		Never		Total	
		*N*	%	*N*	%	*N*	%	*N*	%	*N*	%	*N*	%
Replace	Ortho	80	27.5	77	26.5	79	27.1	30	10.3	25	8.6	291	100
	Non‐ortho	2	0.7	35	11.5	64	21.1	91	29.9	112	36.8	304	100
	Total	82	13.8	112	18.8	143	24.0	121	20.3	137	23.1	595	100
		*χ* ^2^ = 158, *p* < 0.01.^a^											
Repair	Ortho	84	29.2	105	36.5	77	26.6	12	4.2	10	3.5	288	100
	Non‐ortho	35	11.9	62	21.2	51	17.4	82	28.0	63	21.5	293	100
	Total	119	20.5	167	28.7	128	22.0	94	16.2	73	12.6	581	100
		*χ* ^2^ = 118.6, *p* < 0.01.^a^											

Abbreviations: *χ* ^2^, Chi‐square; BR, bonded retainer; GDP, general dental practitioner; N, number; Non‐ortho, GDPs who do not offer orthodontic services; Ortho, GDPs who offer orthodontic services.

^a^ *χ* ^2^ test comparing ortho and non‐ortho according to almost/mostly versus rarely/never.

Table 4 shows that most respondents, regardless of whether they carried out orthodontic treatment, advised that their patients wear their retainers indefinitely. Chi‐squared tests showed that respondents who offered orthodontic services were more likely to advise indefinite wear of RRs (*χ* ^2^ = 15.44, *p* < 0.01) and BRs (*χ* ^2^ = 7.17, *p* < 0.01) than those who did not.

**Table 4:** Advice given by GDPs/belief when patients can stop wearing their retainers.

Retainer type		Wear protocol									
	GDP category	Wear indefinitely		Stop ≤ 2 yrs after orthodontic treatment		Stop ≤ 1 yr after orthodontic treatment		Other		Total	
		*N*	%	*N*	%	*N*	%	*N*	%	*N*	%
RR	Ortho	218	80.4	40	14.8	5	1.8	8	3.0	271	100
	Non‐ortho	196	71.8	51	18.7	11	4.0	15	5.5	273	100
	Total	414	76.1	91	16.8	16	2.9	23	4.2	544	100
BR	Ortho	205	75.9	32	11.9	4	1.5	29	10.7	270	100
	Non‐ortho	179	65.1	52	18.9	14	5.1	30	10.9	275	100
	Total	384	70.5	84	15.4	18	3.3	59	10.8	545	100

Abbreviations: %, percentage; ≤, less than or equal to; BR, bonded retainer; GDP, general dental practitioner; N, number; Non‐ortho, GDPs who do not offer orthodontic services; Ortho, GDPs who offer orthodontic services; RR, removable retainer; Yrs, years.

Table 5 shows that between 57.8% and 61.2% of respondents were very or somewhat concerned that BRs may be associated with periodontal problems over the long term. There was little difference (*χ* ^2^ = 0.59, *p* = 0.44) between those GDPs who offered orthodontic services and those who did not.

**Table 5:** Concern that BRs may be associated with periodontal harm over the long term.

GDP category	Concern											
	Very		Somewhat		A little		Not		Other		Total	
	*N*	%	*N*	%	*N*	%	*N*	%	*N*	%	*N*	%
Ortho	38	14.1	118	43.7	66	24.4	42	15.6	6	2.2	270	100
Non‐ortho	59	21.0	113	40.2	73	26.0	31	11.0	5	1.8	281	100
Total	97	17.6	231	41.9	139	25.2	73	13.3	11	2.0	551	100

Abbreviations: %, percentage; BR, bonded retainer; GDP, general dental practitioner; N, number.

**Figure 2:**
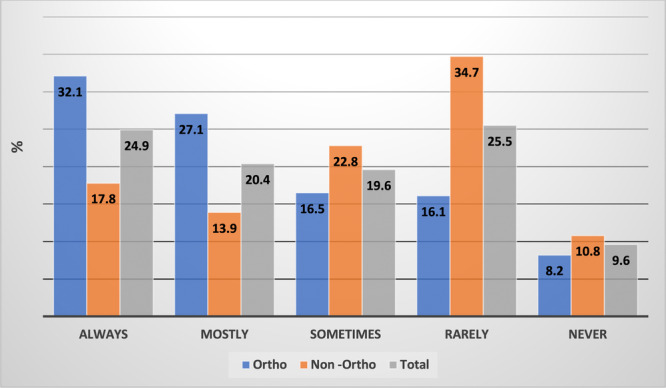
Advice given to patients about bringing their RRs to check‐up or treatment appointments by % GDP (*n* = 514). Key. GDP, general dental practitioner. N, number. Non‐ortho: GDPs who do not offer orthodontic services. Ortho: GDPs who offer orthodontic services. RR: removable retainer.

Respondents who offered orthodontic services were significantly more likely (*χ* ^2^ = 37.5, *p* < 0.01) than those who did not to advise patients to bring their RRs to check‐up or treatment appointments (Figure 2).

Most (*n* = 445; 83.0%) respondents, on the completion of orthodontic treatment by an orthodontist, reported that they would like the orthodontist to inform them that treatment has been completed. A Chi‐squared test (*χ* ^2^ = 45.14, *p* < 0.01) indicated that the number of respondents who did not offer orthodontic services (*n* = 258; 93.8%) and who would like the orthodontist to inform them that treatment had been completed was greater than those who did offer orthodontic services (*n* = 187; 71.6%).

A total of 70.2% (*n* = 374) of respondents indicated that they would like to be informed by the treating orthodontist about patients' retention protocols, with no difference (*p* = 0.45) being recorded between those who offered orthodontic services (*n* = 177; 68.1%) and those who did not (*n* = 197; 72.2%).

Table 6 indicates that GDPs who graduated as a dentist more recently were more likely to consider the retention phase to be indefinite and that GDPs who provided orthodontic services were more likely to always repair and replace retainers compared with those who did not.

**Table 6:** Overview of statistically significant predictors of responses to selected questions following univariable/multivariable binary logistic regression.

	Gender^a^	Years since graduating^b^	Practice setting^c^	Ortho vs. non‐ortho^d^
	OR (95% CI)	OR (95% CI)	OR (95% CI)	OR (95% CI)
Always advise/believe patients wear their RRs indefinitely	*p* > 0.05	*p* < 0.05 0.84 (0.78–0.90)	*p* > 0.05	*p* > 05
Always replace RRs in the event of detachment/breakage	*p* > 0.05	*p* > 0.05	*p* < 0.05 0.72 (0.64–0.77)	*p* < 0.05 0.65 (0.61–0.68)
Always repair RRs in the event of detachment/breakage	*p* > 0.05	*p* > 0.05	*p* > 0.05	*p* < 0.05 0.84 (0.81–0.89)
Always advise/believe patients wear their BRs indefinitely	*p* > 0.05	*p* < 0.05 0.91 (0.88–0.94)	*p* > 0.05	*p* > 0.05
Always replace BRs in the event of detachment/breakage	*p* > 0.05	*p* > 0.05	*p* < 0.05 0.79 (0.69–0.79)	*p* < 0.05 0.65 (0.61–0.68)
Always repair BRs in the event of detachment/breakage	*p* > 0.05	*p* > 0.05	*p* > 0.05	*p* < 0.05 0.73 (0.70–0.78)
Concerned that BRs may be associated with periodontal problems over the long term	*p* > 0.05	*p* > 0.05	*p* > 0.05	*p* > 0.05

*Note: p* > 0.05: non‐significant.

Abbreviations: BR, bonded retainer; OR, odds ratio; CI, confidence interval; RR, removable retainer.

^a^ Female (reference) compared to male.

^b^ Per additional category of year since graduation.

^c^ Private practice setting (reference) compared to others.

^d^ GDPs who offer orthodontic services (ortho) (reference) compared to those who do not (non‐ortho).

The kappa value for test*–*retest survey reliability was 0.94 to 0.97, indicating excellent reliability.

## 4 Discussion

This is the first comprehensive survey of GDP practices and opinions concerning orthodontic retention and retainers in Australia. It is also the first survey to determine the opinions of GDPs based on whether they offered orthodontic services, or not. That orthodontic treatment is a routinely undertaken treatment and that the retention phase is indefinite highlights the importance of the present study. The findings indicated that just less than half of the respondents offered limited or comprehensive orthodontic services and that the CPR combined with the BR was the preferred retainer of GDPs who provided orthodontic treatment in Australia. The findings also suggested that there were differences in opinion regarding the management of the retention phase between the two cohorts and that the orthodontic and dental profession needs to reflect on how best effective management of the retention phase can be applied.

The responses of 683 GDPs were evaluated in the present study. This contrasted with a range of 56 to 502 respondents received in investigations concerning GDPs and orthodontic retention in the Netherlands (Schreuder et al. 2025), Switzerland (Habegger et al. 2017), France (Rafflenbeul et al. 2021), and the United Kingdom (Molyneaux et al. 2021; Kotecha et al. 2015). It was also greater than the 100–382 dentists who responded in recent surveys of GDPs in Australia (Meade and Weir 2024; Khanna et al. 2025; Teoh et al. 2019). This approximated to 5.4% of the non‐student membership of the ADA which compared with 2%–61% recorded in corresponding surveys (Schreuder et al. 2025; Rafflenbeul et al. 2021; Habegger et al. 2017; Kotecha et al. 2015; Meade and Weir 2024; Khanna et al. 2025; Teoh et al. 2019). There are little available data on the percentage number of GDPs who offer orthodontic services although a recent study (Meade and Dreyer 2022) in Australia suggested that up to 91.3% of general dental practices offered CAT as a treatment option. The present investigation found that 47.8% of the respondents offered limited or comprehensive orthodontic services. This compared with the findings of a recent survey (Meade and Weir 2024) of GDPs in Australia in which 165 out of 264 (62.5%) respondents provided CAT and 57 of the 236 (24%) respondents in an investigation of GDP opinions related to orthodontic retention in the Netherlands (Schreuder et al. 2025).

A total of 74.8% of respondents considered that the pre‐treatment situation was a majorly or moderately important factor in the choice of retainer. This corresponded with the findings of studies in Australia (Meade and Dreyer 2019), Ireland (Meade and Millett 2013) and the Netherlands (Renkema et al. 2009) in which it was the factor that most influenced 74%–92.7% of the orthodontists' choice of retainer. It was notable that just 20% of respondents considered third molars to be a majorly or moderately important factor in the choice of retainer. This aligned with the findings in other countries and corresponded with current evidence regarding the minimal role that third permanent molars play in the development of tertiary crowding of the lower incisors (Meade and Millett 2013; Meade and Dreyer 2019; Renkema et al. 2009; Harradine et al. 1998).

The most commonly prescribed retainer in the maxilla and mandible following FAT and CAT provided by the GDPs who offered orthodontic services was the CPR and BR combined. This contrasted with the findings regarding preferred retainer by orthodontists in many cross‐sectional surveys (Padmos et al. 2019; Meade and Millett 2013; Carneiro et al. 2022; Meade and Dreyer 2019; Saw et al. 2025; Al‐Moghrabi et al. 2024; Pratt et al. 2011) in which either a BR or RR was reported to be most frequently prescribed. The use of a RR in addition to a BR may have reflected increased awareness among clinicians of incisor malalignment following bond failure of BRs and the desire to minimize the risk of potential periodontal damage resulting from “Wire Syndrome” (Abu Arqub et al. 2023; Kučera et al. 2022; Katsaros et al. 2007; Charavet et al. 2022; Cornelis et al. 2022). The latter point may have contributed to the 60% of respondents in the present survey who opined that they were “very” or “somewhat” concerned that BRs may be associated with periodontal harm over the long term. It also contrasted with the opinion of GDPs who did not provide orthodontic services in the present survey that the CPR alone was the retainer most effective at maintaining end of orthodontic treatment results.

Between 49.5% and 66.7% of GDPs who did not offer orthodontic services were “rarely” or “never” prepared to repair or replace BRs in the event breakage or loss. This was similar to findings of GDPs in France (Rafflenbeul et al. 2021) and the United Kingdom (Molyneaux et al. 2021; Kotecha et al. 2015) but less than the apparent preparedness of GDPs to undertake repair and replacement of BRs in the Netherlands (Schreuder et al. 2025) and Switzerland (Habegger et al. 2017). Proposed reasons for the low numbers of GDPs prepared to undertake these procedures include financial and time constraints, insufficient undergraduate education and a lack of relevant knowledge (Molyneaux et al. 2021; Kotecha et al. 2015; Arnold et al. 2014). A consideration of these factors is essential if greater involvement of GDPs in the management of BRs is to occur.

The findings of the present study indicated that there is a general acceptance by the respondent GDPs that an indefinite wear of retainers is required to minimize the risk of relapse. The 70.5%–76.1% of respondents who advocated indefinite wear was similar to the rates observed among orthodontists in Ireland (Meade and Millett 2013) and United Kingdom (Singh et al. 2009) although it was less than the 85% or greater observed by orthodontists in more recent investigations in the Netherlands (Padmos et al. 2018), Canada (Carneiro et al. 2022), and Australia (Meade and Dreyer 2019).

The requirement for adherence with lifetime wear of retainers prompts the issue of retention monitoring and retainer maintenance over the short and long term. Several studies (Carneiro et al. 2022; Meade and Dreyer 2019; Saw et al. 2025; Pratt et al. 2011; Singh et al. 2009; Andriekute et al. 2017) reported that most orthodontists carried out retention checks for a period of just up to 3 years following the cessation of active orthodontic treatment. In addition, a 2019 clinical practice guideline on orthodontic retention recommended that patients be referred to the GDP for aftercare in a “systematic and responsible manner” (Wouters et al. 2019). That 28.9%–50.6% of orthodontists in recent national surveys (Carneiro et al. 2022; Meade and Dreyer 2019) have reported concerns about GDPs managing RR and BR checks, however, introduces uncertainty regarding optimal patient care during the retention phase over the longer term. Considered reflection by the dental and orthodontic profession regarding who oversees and how the retention phase is managed is required to ensure effective and pertinent shared decision making, valid consent and optimal patient care (Lasance et al. 2020; Meade et al. 2019; Al‐Moghrabi, Barber et al. 2021; Crory 2018). This may require the introduction of further education in undergraduate dentistry programs and relevant postgraduate courses (Molyneaux et al. 2021; Rafflenbeul et al. 2021; Kotecha et al. 2015; Arnold et al. 2014).

That further education would be beneficial to the respondents was reflected by just 45.3% of respondents reporting that they always or mostly advised their patients to bring their RRs to the check‐up and treatment appointments. This practice characteristic does not appear to have been previously investigated although a UK study (Molyneaux et al. 2021) found that 71.4% of GDPs reported that patients hardly ever or never brought their RRs to be reviewed at general dental check‐up appointments. CPRs constituted the majority of the RRs prescribed by orthodontists and rely on tooth dimensions to be unchanged to maintain fit (Padmos et al. 2019; Meade and Millett 2015; Carneiro et al. 2022; Meade and Dreyer 2019; Saw et al. 2025). It is important, therefore, that the GDP can consider RR wear in the planning of dental intervention and check retainer fit following restorative procedures.

More than four out of five (83%) respondents indicated that they would like the orthodontist to send correspondence advising that treatment had been completed. This compared with 84.6% respondents who similarly replied in a survey of GDPs in Switzerland (Habegger et al. 2017). The responses to surveys in the United Kingdom and Switzerland indicated that 42%–45% of orthodontists provided this correspondence (Molyneaux et al. 2021; Habegger et al. 2017). It is currently unknown, however, how frequently this correspondence occurs in Australia. Future research should aim to determine how commonly orthodontists communicate with GDPs at the end of active treatment and the preferred mode through which this should occur.

Just over 70% (70.2%) of the GDPs responded that they would like the orthodontist to inform them regarding their patient's retention protocol which aligns with the findings of many studies investigating GDP opinions internationally (Molyneaux et al. 2021; Rafflenbeul et al. 2021; Habegger et al. 2017). That GDPs have found that orthodontists do not routinely do this in many countries suggests that this may be a more global issue (Schreuder et al. 2025; Molyneaux et al. 2021; Rafflenbeul et al. 2021; Kotecha et al. 2015). That the orthodontic profession be more diligent in the provision of guidance regarding the management of patients in the retention phase is likely to be a recommendation for the profession in Australia.

The limitations of the present study should be acknowledged. The membership of the ADA numbers approximately 25,000 which constitutes a response rate in the region of 2.8%. This risks a significant non‐response bias. However, the number of respondents is higher than most surveys in this domain. In addition, it was greater than the minimum sample size of 379 based on a population of 25,000 members of the ADA, and a margin of error of 5% with a 95% confidence interval (Khanna et al. 2025). Furthermore, consideration of survey length was necessary to maximize the number of responses (Meade and Millett 2013). Additional questions would have provided more data but potentially have risked lowering the response rate.

With studies (Rafflenbeul et al. 2021; Kotecha et al. 2015; Lasance et al. 2020) indicating that patients and the dental profession believe that the orthodontist is primarily responsible for maintaining orthodontic treatment stability, it is incumbent on the profession to develop detailed guidance and educational information regarding the management of the retention phase (Alkadhimi and Sharif 2019). This will help ensure that valid consent and shared decision‐making processes are followed and patient care is optimal (Rafflenbeul et al. 2021; Lasance et al. 2020; Meade et al. 2019; Al‐Moghrabi, Barber et al. 2021).

## 5 Conclusions

The findings of the present survey indicated that almost half of the respondent GDPs offered orthodontic services and that the CPR and the BR combined was the most prescribed retainer by GDPs following CAT and FAT. Most respondents, regardless of whether they offered orthodontic treatment, advised that their patients wear their retainers indefinitely.

Between 57.8% and 61.2% of respondents were very or somewhat concerned that BRs may be associated with periodontal problems over the long term. Respondents who offered orthodontic services were significantly more likely, than those who did not, to recommend patients bring their RRs to check‐up or treatment appointments. However, between 68.1% and 72.2% reported that they would like to be informed by the treating orthodontist about patients' retention protocols. These findings suggest that it is incumbent on the profession to develop detailed guidance and educational information to GDPs regarding the management of the retention phase.

## Author Contributions

**Maurice J Meade:** conceptualization, methodology, validation, formal analysis, resources, data curation, writing — original draft preparation, writing — review and editing, visualization, project administration. **Craig Dreyer:** formal analysis, vizualisation, writing — review and editing. Both authors have read and agreed to the published version of the manuscript.

## Funding

The authors received no funding for this work.

## Ethics Statement

The study was approved by the University of Adelaide Human Research Ethics Committee [H‐2023‐294].

## Consent

Informed consent was obtained from all subjects involved in the study.

## Conflicts of Interest

The authors declare no conflicts of interest.

## Data Availability

The data that support the findings of this study are available from the corresponding author upon reasonable request.
